# Whole-Genome Sequence of the Nitrogen-Fixing Symbiotic Rhizobium *Mesorhizobium loti* Strain TONO

**DOI:** 10.1128/genomeA.01016-16

**Published:** 2016-10-06

**Authors:** Yoshikazu Shimoda, Hideki Hirakawa, Shusei Sato, Kazuhiko Saeki, Makoto Hayashi

**Affiliations:** a Division of Plant Sciences, National Institute of Agrobiological Sciences, Tsukuba, Ibaraki, Japan; b Department of Technology Development, Kazusa DNA Research Institute, Kisarazu, Chiba, Japan; c Department of Biological Sciences and Kyousei Science Center for Life and Nature, Nara Women’s University, Kitauoya Nishimachi, Nara, Japan

## Abstract

*Mesorhizobium loti* is the nitrogen-fixing microsymbiont for legumes of the genus *Lotus*. Here, we report the whole-genome sequence of a *Mesorhizobium loti* strain, TONO, which is used as a symbiont for the model legume *Lotus japonicus*. The whole-genome sequence of the strain TONO will be a solid platform for comparative genomics analyses and for the identification of genes responsible for the symbiotic properties of *Mesorhizobium* species.

## GENOME ANNOUNCEMENT

*Mesorhizobium loti* is the symbiont for several *Lotus* species, and strain MAFF303099 is the first nitrogen-fixing rhizobial species whose complete genome was sequenced (1). The *M. loti* strain TONO, together with the strain MAFF303099, has been used widely as a symbiont for a model legume, *Lotus japonicus* (2–6), and these two strains exhibit different symbiotic properties on certain symbiotic mutants of *L. japonicus* (Umehara Y and Shimoda Y, unpublished data). Here, we present the whole-genome sequence of the *M. loti* strain TONO for future study of the rhizobial genes responsible for the symbiotic properties of *Mesorhizobium* species.

The genome of *M. loti* strain TONO was sequenced using Roche 454 GS-FLX Titanium, and a total of 637.1 Mbp (~75-fold coverage) sequence data were obtained from single and paired-end libraries. Obtained reads were assembled by Newbler Assembler (454 Life Sciences), generating eight scaffolds. Sequences of gapped regions inside scaffolds and connections among the eight scaffolds were determined by PCR amplicon sequencing and direct sequencing of TONO genomic DNA by using the same procedures reported in Shimoda et al. (7). The genome of TONO was finally composed into four circular replicons, a chromosome (7,856,088 bp, 62.8% G+C content) and three plasmids (294,703 bp with 59.5% G+C content, 220,869 bp with 58.9% G+C content, and 80,491 bp with 60.4% G+C content). Genome annotation was performed using the Microbial Genome Annotation Pipeline (MiGAP) provided by DDBJ (8). A total of 8,149 protein-coding genes, 57 tRNAs, and six rRNAs (two copies of 16S-23S-5S operon) were predicted on the genome. Approximately 83% of protein-coding genes (6,774 genes) are assigned to Clusters of Orthologous Groups (COGs) (9).

We compared the genome components of the strain TONO with those of closely related genome of the *M. loti* strains MAFF303099 (1) and R7A (10). Average nucleotide identity (ANI) (11) values between TONO and MAFF or R7A are 91.7% and 91.5%, respectively. In our assembled TONO genome, a 508-kb symbiotic island integrated into a phenylalanine-tRNA gene with the duplication of 3′ terminal portion of the gene was predicted. The symbiotic island of TONO carries genes involved in Nod factor synthesis (*nod* genes) and nitrogen fixation (*nif* and *fix* genes), and these gene clusters are highly conserved among the three strains in their sequence and order of genes. Also, gene clusters encoding the components of a type III secretion system and conjugative transfer proteins are highly conserved between TONO and MAFF303099.

Dot plot similarity analysis of overall TONO chromosome with that of MAFF303099 and R7A revealed that these three strains share collinear similarity throughout the chromosome, except for symbiotic islands, which show fragmented similarity. Furthermore, a comparison of the predicted gene products revealed that approximately 10% of the predicted TONO genes (834 genes) did not show significant similarity (e-value <10^-4^) to any genes of MAFF303099 and R7A, and the majority of these genes show similarity to functionally uncharacterized proteins of other bacteria. Although further analyses are needed to identify the genes responsible for the differences in symbiotic properties of *Mesorhizobium loti*, the whole-genome sequence of strain TONO we reported here provides a solid platform for future studies in *Mesorhizobium* species.

### Accession number(s).

The genome sequences have been deposited at DDBJ/EMBL/GenBank under the accession numbers AP017605 to AP017608.
